# Energy-consuming right transaction and low-carbon technology innovation: Evidence from Chinese listed enterprises

**DOI:** 10.1016/j.heliyon.2024.e36277

**Published:** 2024-08-14

**Authors:** Jingchan Wang, Wei Chen

**Affiliations:** a Wuchang Shouyi University, Wuhan, 430064, China; b School of Public Administration, Zhongnan University of Economics and Law, Wuhan, 430073, China

**Keywords:** Energy-consuming right transaction, Low-carbon technology innovation, R&D personnel investment, Green agency cost, Digital finance

## Abstract

To decarbonize the economy and spur high-quality development, formulating effective environmental policies to encourage low-carbon technology innovation is increasingly critical. While the energy-consuming right transaction represents a significant institutional breakthrough, its potential to motivate enterprises towards low-carbon technology innovation remains underexplored. To address this gap, our study utilizes panel data from Chinese listed enterprises between 2009 and 2020, employing the energy-consuming right transaction pilot policy to develop a difference-in-difference model that assesses the policy's impact on low-carbon innovation. Our findings indicate that the implementation of energy-consuming rights transaction has boosted low-carbon innovation efforts by 14.3 %. In-depth analysis shows that R&D investment and green agency costs are crucial mediators, with energy-consuming rights transaction enhancing R&D capital and personnel investments by 2.1 % and 1.5 %, respectively, and reducing green agency costs by 1.8 %. The study also uncovers the moderating role of digital finance, which amplifies the positive effects of energy-consuming rights transaction on low-carbon innovation. Moreover, energy-consuming rights transaction shows a more significant effect on improving low-carbon innovation for low energy-consuming and non-state-owned enterprises. These insights underscore the importance of precisely segmenting energy-consuming enterprises and devising customized policies to meet their unique needs, paving the way for a national energy-consuming right transaction market.

## 1 Introduction

The high energy consumption development model is the biggest challenge of China's commitment to achieving carbon peak and carbon neutrality. Since 2015, China's annual carbon emissions have consistently risen, surpassing 10.5 billion tons. Factors like the Russia–Ukraine war in 2021 have driven up natural gas prices, causing a strong recovery in coal-fired power generation and a sharp rebound in global energy demand.^1^ China's total carbon emissions have rose sharply to 12.04 billion tons in2021.^2^ To meet the its commitment within a short time frame, China needs to quickly decarbonize its energy structure. Previous research has highlighted the effectiveness of low carbon technology innovation (LCTI) in reducing carbon emissions [1]. LCTI, which encompasses a range of technologies primarily focused on renewable energy, acts as a catalyst for reducing carbon emissions by optimizing the energy landscape. LCTI stands as a pivotal element in promoting sustainable, environmentally friendly business practices [2]. By streamlining energy utilization and encouraging more efficient allocation of energy resources to curtail carbon emissions, LCTI not only lightens the load of green transformation for enterprises but also plays a vital role in addressing energy and environmental challenges, thereby fostering green economic growth [3,4].

To advance green development, China has shifted from traditional end-stage sulfur dioxide and carbon emissions trading to a more proactive approach with the Energy-Consuming Right Transaction (ECRT) policy. Initiated in 2017 in provinces like Zhejiang and Fujian, ECRT enables companies to manage and trade their total energy consumption rights more effectively. This upstream control strategy aims to reduce overall energy usage, encourage eco-friendly business transformations, and promote efficient resource allocation, distinguishing it from conventional emission trading systems focused on pollution management.

This paper aims to clarify the relationship between ECRT and LCTI. The Porter Hypothesis posits that suitable environmental regulation can stimulate technological innovation [5]. Studies have found that regulation compliance costs can either lead to a mere financial burden or incentives to innovate, depending on policy design [4,6]. However, most studies focus on the impact of end-of-life regulation tools on innovation [2,3,7], while research on the impact of upstream control settings is limited. Different from end-of-life regulations, ECRT involves multiple objectives such as energy conservation, emissions reduction, and improving energy efficiency. And firms may have to restructure their energy use at the beginning of production. Therefore, firms may under greater regulatory compliance pressure. Whether upstream control tools will lead a favorable result regarding with low carbon innovation is unanswered but important question. We leverage ECRT as a quasi-natural experiment to assess its impact on enterprise LCTI through a difference-in-differences (DID) model. By comparing the changes in LCTI after the policy for treated group (enterprises in pilot areas) and control group (enterprises not in pilot areas), we find that ECRT has a significantly positive impact on LCTI. We also further investigate the mechanisms at play and the characteristics of this effect. Investigating the impact of ECRT on LCTI is vital for verifying the applicability of the Porter Hypothesis in the Chinese context. Additionally, elucidating the linkage between ECRT and LCTI carries practical implications for advancing sustainable development and policy promotion within China's energy trading system, as seen from the perspective of LCTI. Existing studies have explored the role of policy stringency [2], financial constraint [3], and innovation cooperation [8]. However, the inner mechanism inside the firm remains unclear. Our goal, therefore, is to reveal the channels at work within enterprises. Therefore, this study also uncovers the impact mechanisms of ECRT on LCTI from three angles: R&D capital investment (RDC), R&D personnel investment (RDP), and green agency cost (GAC). Moreover, it examines the moderating influence of digital finance (DF), which is growingly important in helping firms easing financial constraint, but have not yet received enough attention from literature [9].

This study offers several contributions. First, it contributes to the existing literature by examining the effect of ECRT on LCTI, thereby enriching our understanding of the policy implications of ECRT and corporate innovation. It supplements the available micro-level evidence and addresses gaps in prior research. Second, it dissects the internal mechanisms driving the promotion of LCTI by ECRT, incorporating RDC, RDP, and GAC. Although abundance of literature investigates Porter Hypothesis in regard to innovation, it remains unclear how companies improve their LCTI under the pressure of environmental mechanism. Third, it identifies, for the first time, the moderating effect of DF on the ECRT–LCTI nexus. Last, it enriches heterogeneity analyses related to the relationship between ECRT and LCTI. Notably, this study explores the heterogeneity in enterprises’ knowledge assets and financing constraints, providing both theoretical explanations and empirical evidence to bolster high-quality economic development driven by ECRT. These findings carry substantial practical and policy implications.

## 2 Literature review and research hypothesis

Unlike environmental regulations focusing on carbon emissions, ECRT targets the root cause: energy consumption. This shift signifies the Chinese government's move from endpoint to upstream control, aligning with its broader agenda to overhaul the energy sector. Given China's propensity to scale pilot policies nationwide, comprehending ECRT's mechanisms and impacts is crucial.

The ECRT market functions effectively due to the limited availability of energy-consuming rights, with the government annually allocating fixed quotas to businesses. Enterprises must buy additional quotas in the ECRT market if their consumption exceeds these limits to avoid penalties. Conversely, underutilization allows them to sell surplus quotas for profit. Operating like carbon trading systems, the ECRT uses these quotas as market assets to regulate energy usage, facilitating energy conservation, emission reduction, and low-carbon transformation.

The implementation of ECRT has sparked scholarly interest, leading to emerging research that investigates its environmental and economic ramifications, albeit in limited scope. Wang et al. [10] demonstrates that China's Energy-Consuming Rights Trading significantly enhances firms' Green Total Factor Productivity, mainly through encouraging green technological innovations and improving capacity and financial resource allocation. Zhou et al. [11] use a difference-in-difference model to evaluate how the ECRT impacts energy efficiency in Chinese listed firms, particularly benefiting state-owned enterprises, high-pollution companies, and those in regions with scarce resources, ultimately increasing their market value. Wang et al. [12] evaluate the policy effect of ECRT and find that ECRT can promote pilot areas to reduce carbon emissions and increase economic profits, thereby reducing carbon emission intensity. Industrial upgrading has a positive effect on China's carbon emissions reduction, indicating that enhancing the level of industrial upgrading should become an effective means for China's current emission reduction and carbon reduction.

The similarity between the EU's White Certificate System (WCS) and China's ECRT policy enriches the literature, enhancing our understanding of ECRT. In 2005, the Italian government officially established the WCS. The UK, France, Denmark, and the Flanders region of Belgium have successively implemented this policy. They have subsequently confirmed the superiority of the WCS and its effectiveness as an energy-saving and emission reduction tool. Research on the WCS mainly revolves around the following three aspects. In terms of the energy-saving effect of WCS, Oikonomou et al. [13] show that the WCS can stimulate market parties to implement innovation, improve energy efficiency, and identify energy-saving opportunities. Oikonomou et al. [14] later point out that the WCS can improve the energy efficiency of power suppliers. Friedrich et al. [15] believe that the WCS is a mechanism that can promote the development of energy efficiency markets and improve demand side energy efficiency. Franz et al. [16] believe that the WCS is a successful example of improving industrial energy efficiency. Khatoon et al. [17] study the benefits and key influencing factors of introducing blockchain technology in the WCS for improving energy efficiency.

In terms of the environmental effects of the WCS, Hamrin et al. [18] point out that the WCS essentially represents the environmental benefits brought by energy conservation. Child et al. [19] explore the interaction between the WCS and other emission reduction policy tools in Europe, believing that the WCS can promote the effectiveness of existing policy tools. Oikonomou et al. [20] study the possible synergistic effects between the WCS and the joint compliance mechanism as well as the carbon offsetting system. The study shows that the policy system combining the WCS and the joint compliance mechanism is efficient from the perspective of achieving specific emission reduction goals. Meran and Wittmann [21] point out that a market-based WCS should be put on the agenda in several European countries to reduce carbon emissions and further increase green energy production. Petrella and Sapio [22] believe that the trading mechanism of the WCS has significant volatility, but it still achieves the goal of improving the environment in an imperfect market environment.

In terms of the cost effect of the WCS, Suerkemper et al. [23] point out that the trading part of the WCS aims to improve the cost effect, that is, to achieve mandatory energy-saving goals at the lowest cost. Mundaca [24] analyzed the significance of the WCS for households and the business sector based on three evaluation indicators (cost effect, environmental effect, and distribution fairness). Their findings reveal the significant cost effects of the WCS. Perrels [25] believes that compared with energy taxes, the WCS can stimulate energy-saving potential at the lowest cost. Transue and Felder [26] find that the WCS can reduce harmful emissions without increasing upfront costs and energy consumption.

Regrettably, as the innovative ECRT policy is relatively new, no literature specifically addresses the impact of ECRT on enterprise LCTI, a key factor in mitigating climate change and promoting sustainable development. Investigating ECRT's potential to enhance LCTI is therefore crucial. Despite limited direct research, existing studies offer insights into the relationship between ECRT and enterprise LCTI. We hypothesize that the ECRT system positively impacts enterprise LCTI.

**First**, the relationship between ECRT and LCTI relates to a broad literature investigating the influence of environmental regulation on innovation. Porter [27] introduces the concept of enterprise innovation into environmental policy discussions and, through case studies, demonstrates that appropriate ER, especially market-oriented approaches, can incentivize businesses to engage in green innovation. This leads to the development of markets for environmentally friendly products and technologies, offsets environmental costs, and fosters a harmonious relationship between the economy and the environment, a concept known as the Porter Hypothesis. To assess the applicability of the Porter Hypothesis in China, scholars analyze the impact of China's ER on enterprise LCTI. Zhu et al. [2] conduct empirical tests on the effect of China's carbon trading policy on enterprise LCTI, concluding that carbon trading motivates enterprises to enhance LCTI through market-oriented mechanisms. Subsequently, Qi et al. [3] and Zhao et al. [4] arrive at similar conclusions. Chen et al. [6] examines the influence of green credit policies on LCTI and find that such policies can enhance management efficiency by guiding capital allocation, thereby promoting the level of enterprise LCTI. Qu et al. [7] observe that China's low-carbon city policies have increased the quantity of LCTI initiatives among enterprises, although the quality of these initiatives has not necessarily improved. The literature thus provides a reasonably comprehensive understanding of the impact of environmental regulation on enterprise LCTI.

It is possible that Porter Hypothesis applies to the settings of ECRT. The research on carbon market shed light on the relationship between market-based environmental regulation and LCTI. Researches on carbon markets in general conclude that this cost pressure induces firms to innovate. Lyu et al. [28] use a DID model to find that the carbon emission trading mechanism inhibits LCTI in the short term. Most scholars, however, are of the view that this mechanism has a significant role in promoting LCTI [[29], [30], [31]]. Some argue that raising carbon trading prices and carbon trading tax rates also promote LCTI [32]. Drawing on further analysis of LCTI classification, Zhang [33] demonstrates that carbon market liquidity can also promote regional LCTI through breakthrough innovation and incremental innovation.

Similar to carbon trading, the market-driven ECRT system imposes compliance costs due to government-imposed limits on annual energy consumption. Enterprises must adopt low-carbon processes or maintain production levels within these limits, facing extra costs for exceeding quotas. Reducing production to cut energy costs can decrease operating income and competitiveness. Therefore, the ECRT policy pressures enterprises to save energy and enhance production efficiency, leading to increased investment in low-carbon technology innovation (LCTI) to reduce energy use, cut costs, and potentially increase profits.

**Second**, profit from low-carbon technology innovation (LCTI) boosts firms’ motivation to develop green technologies. Growing environmental awareness, driven by increased public and social focus on sustainability issues [34,35], encourages firms to adopt sustainable practices. LCTI not only curtails energy consumption but also enables firms to profit from selling excess energy quotas, enhancing their economic benefits. This process, known as innovation compensation, helps firms enhance production efficiency and profitability, balancing out the costs of energy rights. Thus, the ECRT incentivizes firms to pursue LCTI, fostering a cycle of innovation and profit enhancement.

Thus, this study proposes the following hypothesis:

H1 Compared with unaffected enterprises, the implementation of ECRT will increase the LCTI level of affected enterprises.

To unravel the mechanisms underpinning the relationship between Energy Consumption Rights Trading (ECRT) and Low-Carbon Technology Innovation (LCTI), this study draws on existing literature to examine the roles of R&D capital investment, R&D personnel investment, and green agency costs. Unlike internal mechanisms that firms leverage to boost innovation, digital finance acts as an external facilitator, easing the financial constraints that inhibit innovation. Consequently, the moderating role of digital finance is also scrutinized.

R&D capital investment (RDC) is pivotal in driving green innovation and technological advancements. And the investment in R&D personnel (RDP) is essential for fostering a culture of innovation and technological development within organizations. Lee and Min [36] found that green R&D investment significantly impacts carbon emissions and firm performance. Zhang et al. [37] further support this finding, illustrating how R&D investment, influenced by environmental regulation, propels green technology innovation in China. Duque‐Grisales et al. [38] highlighted the relationship between green innovation and financial performance, noting the moderating role of ISO 14001 and R&D investment. Fan and Teo [39] demonstrated that R&D investment in China positively correlates with green innovation performance, suggesting that RDP plays a critical role in achieving sustainable technological growth.

Accordingly, we hypothesize:

H2 Enhanced R&D capital and personnel investments will positively influence the relationship between ECRT and LCTI.

Green agency cost (GAC) involves expenses related to ensuring that corporate activities align with environmental goals. Holmstrom [40] and Francis and Smith [41] discussed how agency costs could influence innovation. Belloc et al. [42] explored the corporate governance effects on innovation, considering agency costs and asset specificity, highlighting the complexity of managing GAC to promote sustainable innovation. Tang [43] found that ESG performance significantly promotes corporate innovation, which is mediated by alleviating agency cost.

Consequently, we hypothesize:

H3 Lower green agency costs will strengthen the relationship between ECRT and LCTI.

Digital finance has been recognized as a catalyst for innovation, especially in the context of green technology. Feng et al. [44] and Zhang J. et al. [45] both found that digital finance facilitates green technology innovation, with the latter study emphasizing the digital economy's role in driving low-carbon development. Chen [46] investigates the influence of the digital economy on low-carbon innovation among China's A-share listed companies from 2012 to 2019, finding that digital growth alleviates financing constraints and reduces environmental uncertainty, which stimulates sustainable practices. Liu et al. [47] and Wu et al. [48] provided evidence of how digital finance contributes to corporate green innovation and low-carbon development, respectively, showcasing its importance in supporting sustainable technological advancements.

Thus, we propose:

H4 Digital finance positively moderates the relationship between ECRT and LCTI.

## 3 Data and methodology

### 3.1 Estimation strategy

We regard the ECRT policy implemented in China in 2017 as an exogenous policy shock to identify the effect of ECRT on LCTI. Difference-in-Difference (DID) is a frequently used method to estimate the causal effect of a treatment, intervention, or policy change on an outcome variable. The DID method leverages the difference between the changes in the outcome variable for the treatment group (those exposed to the intervention before and after the intervention and the changes in the outcome variable for the control group (those not exposed to the intervention) over the same periods. By comparing these differences, DID attempts to isolate the causal effect of the treatment [49]. Therefore, DID specification is an ideal tool for this study to investigate the treatment effects of ECRT. Referring to literature [12], we establish a DID model to evaluate the effect of ECRT on the LCTI of enterprises.

(1) $${\text{LCTI}}_{it}=\alpha_{0}+\alpha_{1}{\text{Policy}}_{i}\times {\text{Time}}_{t}+\alpha_{2}{\text{CV}}_{it}+\mu_{i}+\gamma_{t}+\varepsilon_{it}$$

In equation (1), *LCTI*_*it*_ represents the LCTI of enterprise *i* in year *t*, and *Ploicy × Time* represents a proxy variable for ECRT policy. Coefficient *α*_*1*_ represents the effect of ECRT on the LCTI of enterprises. *Policy* is a dummy variable of the treatment group representing the enterprises in the pilot areas: 1 is assigned to the treatment group, and 0 is assigned to the rest. *Time* is the time dummy variable, with a value of 1 after the ECRT implementation and 0 before the ECRT implementation. The coefficient between *Policy* and *Time* represents the difference between the changes in LCTI of companies in pilot areas and those that are not affected by the policy. Therefore, $\alpha_{1}$ reflects the policy impacts of ECRT. *CV* represents control variables, and μ and γ represent the individual and year fixed effects, respectively. Individual fixed effects account for time invariant firm specific characteristics; and year fixed effects account for common shocks for all firms in each year, such as national policy changes.

### 3.2 Data and variables

#### 3.2.1 Data source

The data in this study is sourced from the China Stock Market & Accounting Research Database (CSMAR), a major data provider in China. We use the data from A-share listed industrial enterprises in China, covering the period from 2009 to 2020, as our primary sample. The current low awareness of energy-saving practices and inefficient utilization of energy resources within enterprises have considerably impeded China's progress toward low-carbon development. As the main consumers of energy, industrial enterprises bear a great responsibility and obligation to carry out LCTI. Although the ECRT policy does not explicitly target specific industries, the focus of its implementation centers on industrial enterprises. Therefore, we focus our discussion on industrial firms, which are selected based on the industry code provided by CSMAR. Based on enterprises' office addresses, we are able to distinguish between those in ECRT pilot areas and those in non-pilot areas. The former companies are impacted by the policy and hence serve as the treated group, while the latter serve as the control groups. We exclude enterprises categorized as ST (Special Treatment) and ST*, because such firms face financial difficulties and their financial data may be inaccurate or unreliable. We also conduct tail reduction on the continuous variables, eliminating extreme values at the 1st and 99th percentiles to remove interference from outliers. Further excluding observations with missing data on key variables data leaves us with a sample of 21046 observations.

#### 3.2.2 Variable definitions

LCTI: Low-Carbon Technological Innovation (LCTI) refers to pioneering activities where enterprises enhance their carbon reduction, capture, and sequestration technologies at crucial production stages. This innovation targets energy conservation, emission reduction, and better resource utilization. Referring to the research methods of Zhu et al. [2], we use the number of low-carbon patents to measure LCTI.

Control variables (CVs): To accurately estimate the effect of ECRT on LCTI, we identify several critical control variables that may influence the expansion of LCTI, including total assets, age, cash flow, and shareholding ratio, leverage, number of employees, and ROA. Table 1 presents the definition and descriptive statistics of these CVs.

**Table 1:** Descriptive statistics.

Variable	Obs.	Definition	Mean	SD	Min.	Max.
LCTI	21046	Logarithm of the number of low-carbon patents	0.346	0.764	0	6.8
Asset	21046	Logarithm of total assets	21.882	1.214	16	29
Age	21046	Logarithm of company's establishing time	2.440	0.423	0	4
Cashflow	21046	Ratio of operating net cash flow to total assets	21.369	1.488	12	29
Top1	21046	Shareholding ratio of largest shareholder	0.176	0.381	0.003	0.91
Lev	21046	Ratio of liabilities to total assets	0.332	0.211	0.011	1.68
Labor	21046	Logarithm of number of employees	7.013	1.223	2	13
Roa	21046	Ratio of net profit to total assets	0.622	0.424	0.11	0.509

## 4 Analysis of empirical results

### 4.1 DID model results

Based on equation (1), Table 2 reports the results of sequential addition of CV, individual fixed effect, and time fixed effect. The coefficients of *Policy × Time* are positive, indicating that ECRT can encourage the LCTI of enterprises. Column (1) in Table 2 presents the estimated results without control variables and fixed effects. Including control variables and firm fixed effects reduces the magnitude of the coefficient on policy implementation in Column (2), though remain significant in 1 % level. And in Column (3), our preferred specification, we further account for year fixed effects to control year specific common shocks for all firms. The estimated result indicates that after the launch of ECRT, the changes in the number of low carbon patents filed by companies in pilot areas are 14.3 % higher than those in non-pilot areas. The results remain consistently positive and significant across columns, which validates our research hypothesis.

**Table 2:** Effect of NIC on LCI

	(1)	(2)	(3)
	LCTI	LCTI	LCTI
Policy × Time	0.218***	0.159***	0.143***
	(0.043)	(0.026)	(0.023)
Asset		0.083***	0.069***
		(0.0098)	(0.0101)
Age		0.126	0.088
		(0.322)	(0.145)
Cashflow		0.049	0.027
		(0.098)	(0.086)
Top1		−0.0856	−0.0569
		(0.0765)	(0.0611)
Lev		0.011*	0.0097*
		(0.0055)	(0.0057)
Labor		0.005	0.0196**
		(0.008)	(0.0083)
Roa		−0.011	−0.0055
		(0.0101)	(0.009)
ID effect	NO	YES	YES
Year effect	NO	NO	YES
Observations	21046	21046	21046
R-squared	0.029	0.755	0.763

Robust standard errors are in parentheses; *p < 0.1, **p < 0.05, ***p < 0.01. These notes are the same for other tables.

As indicated by the positive coefficient on asset, the total assets of a firm positively influence its low carbon innovation. Every one percentage increase in a firm's total asset will lead to 0.069 % increase in LCTI. Leverage and labor also exert positive impact on LCTI. These results are consistent with economic intuition since firms with larger assets, workforce as well as external source of capital are more capable of investing in research. However, we do not find any significant impacts from age, cash flow, ROA, and the shareholding ratio of the largest shareholder.

### 4.2 Robustness tests

#### 4.2.1 Parallel trend test

The parallel trend hypothesis between the pilot and non-pilot enterprises is the premise to obtain unbiased estimation results by using DID method. We construct the following model to test the parallel trend hypothesis.

(2) $${\text{LCTI}}_{it}=\beta_{0}+\sum_{t=2014}^{2020}\beta_{t}{\text{Policy}}_{i}\times {\text{Time}}_{t}+\beta_{2}{\text{CV}}_{it}+\mu_{i}+\gamma_{t}+\varepsilon_{it}$$

Table 3 reports the test results of equation (2). The DID coefficients of *Policy × Time*_*2014*_*, Policy × Time*_*2015*_*, Policy × Time*_*2016*_ are not significant, indicating that the LCTI of treated firms are not significantly different from control firms. This pattern validates the parallel trend hypothesis. The DID coefficients of *Policy × Time*_*2017*_*, Policy × Time*_*2018*_*, Policy × Time*_*2019*_ and *Policy × Time*_*2020*_ are significant, which means that the significant difference in LCTI between pilot and non-pilot enterprises occurs after ECRT policy implementation. The magnitude of coefficients gets larger along years, indicating that the dynamic effects of the policy build up.

**Table 3:** Parallel trend test results.

	(1)	(2)
	LCTI	LCTI
Policy × Time_2014_	0.114	0.0885
	(0.165)	(0.0899)
Policy × Time_2015_	0.0742	0.06
	(0.0711)	(0.0622)
Policy × Time_2016_	0.0599	0.0457
	(0.0622)	(0.0497)
Policy × Time_2017_	0.049*	0.0612**
	(0.0272)	(0.031)
Policy × Time_2018_	0.0811***	0.0802***
	(0.029)	(0.0291)
Policy × Time_2019_	0.1352***	0.157***
	(0.019)	(0.03)
Policy × Time_2020_	0.124***	0.129***
	(0.027)	(0.026)
CV	NO	YES
ID effect	YES	YES
Year effect	YES	YES
Observations	21046	21046
R-squared	0.732	0.775

#### 4.2.2 Placebo test

While the DID model shows significant improvement in LCTI due to the ECRT policy, further investigation is needed to ensure the results are not influenced by other factors. To this end, we perform a placebo test by adjusting the timing of the policy's implementation to hypothetical years 2014 and 2013, and exclude data from 2017 onward. We then re-estimate equation (1) to examine the counterfactual outcomes. The coefficients of *Policy × Time* in Table 4 are insignificant.

**Table 4:** Placebo test.

	(1)	(2)
	LCTI	LCTI
Policy × Time	0.072	0.054
	(0.096)	(0.084)
CV	NO	YES
ID effect	YES	YES
Year effect	YES	YES
Observations	13030	13030
R-squared	0.722	0.721

#### 4.2.3 Propensity score matching (PSM)–DID

To address potential biases due to differences between pilot and non-pilot enterprises in our study, we applied Propensity Score Matching (PSM). Initially, a Logit regression on the control variables was conducted to generate propensity scores for both groups. We then used the caliper matching method to pair the samples. After these adjustments, the re-estimation of equation (1) showed that the coefficients of *Policy × Time* in Table 5 remained significantly positive at the 1 % level.

**Table 5:** PSM–DID estimation results.

	(1)	(2)
	LCTI	LCTI
Policy × Time	0.138***	0.139***
	(0.0225)	(0.0244)
CV	NO	YES
ID effect	YES	YES
Year effect	YES	YES
Observations	17340	17340
R-squared	0.758	0.757

#### 4.2.4 Adjustment of sample time window

To ensure a consistent analysis period, we adjusted the sample timeframe to span from 2014 to 2019 and subsequently re-estimated equation (1). The results, displayed in Columns 1 and 2 of Table 6—with and without control variables, respectively—confirm that the coefficients of *Policy × Time* remain statistically significant. This consistency in findings across different sample intervals reaffirms the validity of Hypothesis 1.

**Table 6:** Adjustment of the sample time window.

	(1)	(2)
	LCTI	LCTI
Policy × Time	0.177***	0.169***
	(0.0118)	(0.0143)
CV	NO	YES
ID effect	YES	YES
Year effect	YES	YES
Observations	13356	13356
R-squared	0.863	0.824

#### 4.2.5 Exclusion of the effects of ER

Prior research has suggested that China's carbon trading policy (CTP) and low-carbon city policy (LCP) can influence firms' LCTI [2,7]. To ensure the robustness of our results, we accounted for the potential effects of these ERs. In 2013, China initiated the CTP in seven provinces, aiming to incentivize enterprises to develop low-carbon technologies, which could impact their LCTI. To address this, we introduce the cross-multiplication term of province fixed effects and time effects into equation (1) to control for the influence of the CTP. Column 1 of Table 7 reveals that the coefficient of *Policy × Time* is significant. In 2010, China introduced the LCP to promote low-carbon transformation. To account for the potential impact of the LCP, we incorporate the cross-multiplication term of city fixed effects and time fixed effects into equation (1). Column 2 of Table 7 reveals that the coefficient of *Policy × Time* is significant.

**Table 7:** Exclusion of effect of ER.

	(1)	(2)
	LCTI	LCTI
Policy × Time	0.118***	0.113***
	(0.0187)	(0.017)
CV	YES	YES
ID effect	YES	YES
Year effect	YES	YES
Observations	21046	21046
R-squared	0.755	0.755

### 4.3 Mechanism analysis

Having confirmed that the ECRT enhances LCTI, the study progresses to explore if R&D capital investment (RDC), R&D personnel investment (RDP), and Green Agency Cost (GAC) mediate this impact. A new model is constructed to test whether ECRT can boost LCTI through these specific mediatory channels of RDC, RDP, and GAC.

(3) $${\text{Med}}_{it}=\beta_{0}+\beta_{1}{\text{Policy}}_{i}\times {\text{Time}}_{t}+\beta_{2}{\text{CV}}_{it}+\mu_{i}+\gamma_{t}+\varepsilon_{it}$$

(4) $${\text{LCTI}}_{it}=\lambda_{0}+\lambda_{1}{\text{Policy}}_{i}\times {\text{Time}}_{t}+\lambda_{2}{\text{Med}}_{it}+\lambda_{3}{\text{CV}}_{it}+\mu_{i}+\gamma_{t}+\varepsilon_{it}$$

In equations (3) and (4), *Med* is the mediating variable, which include *RDC*, *RDP*, and *GAC*. To measure the mediating factors in our study, we define operational proxies as follows: RDC is represented by the ratio of R&D expenses to operating income; RDP is indicated by the proportion of R&D personnel relative to total employees; and GAC is measured by the ratio of environmental protection management expenses to operating revenue. These proxies will help quantify the mediation effects of these variables on the relationship between ECRT and LCTI. We assess the mediating effects of RDC, RDP, and GAC through the significance of *β*_*1*_, *λ*_*1*_, and *λ*_*2*_.

#### 4.3.1 Mediating effect of RDC

We report the results based on applying equations (3) and (4) to RDC. Columns 1 and 2 of Table 8 indicate that both *Policy × Time* and RDC coefficients are significant, suggesting RDC serves as a mediating factor in the ECRT's impact on LCTI. RDC enhances technological progress by providing financial resources necessary for updating production processes, thereby accelerating LCTI. Furthermore, ECRT policy encourages companies lacking energy rights to buy from those with surpluses, promoting investment in RDC to achieve quicker and more cost-effective LCTI, aligning with China's stringent carbon reduction goals. This mechanism is pivotal in boosting LCTI through enhanced RDC.

**Table 8:** Mechanism analysis.

	(1)	(2)	(3)	(4)	(5)	(6)
	RDC	LCTI	RDP	LCTI	GAC	LCTI
Policy × Time	0.021***	0.118***	0.015*	0.113***	−0.018*	0.109***
	(0.007)	(0.0198)	(0.0085)	(0.0289)	(0.01)	(0.026)
RDC		0.817***				
		(0.144)				
RDP				1.567*		
				(0.865)		
GAC						−0.986*
						(0.538)
CV	YES	YES	YES	YES	YES	YES
ID effect	YES	YES	YES	YES	YES	YES
Year effect	YES	YES	YES	YES	YES	YES
Observations	21046	21046	21046	21046	21046	21046
R-squared	0.511	0.766	0.488	0.769	0.722	0.773

#### 4.3.2 Mediating effect of RDP

Columns 3 and 4 of Table 8 reveal that the Policy × Time and RDP coefficients are statistically significant, indicating RDP as a crucial mediator in the effect of ECRT on LCTI. Quality R&D personnel enhance LCTI by transforming innovative knowledge into tangible innovations, leveraging the incentives provided by ECRT. This underscores the importance of RDP in propelling technological progress and sustainable enterprise development, particularly as enterprises align with China's increasing carbon reduction targets. Hence, ECRT effectively boosts LCTI by enhancing RDP capacities within enterprises. This finding validates our research hypothesis H2.

#### 4.3.3 Mediating effect of GAC

Columns 5 and 6 of Table 8 indicate significant coefficients for *Policy × Time* and GAC, suggesting GAC as a mediator in ECRT's impact on LCTI. ECRT reduces green agency costs, promoting LCTI by aligning shareholder and manager incentives toward minimizing environmental penalties. By increasing the costs of non-compliance, ECRT compels enterprises to prioritize investments in low-carbon technologies, thereby reducing green agency costs and enhancing overall enterprise LCTI. This mechanism confirms the effectiveness of ECRT in fostering sustainable enterprise practices.

### 4.4 Moderating effect of DF

The following model is constructed to test the moderating effect of DF on ECRT and LCTI:

(5) $${\text{LCTI}}_{it}=\lambda_{0}+\lambda_{1}{\text{Policy}}_{i}\times {\text{Time}}_{t}\times {\text{DF}}_{it}+\lambda_{2}{\text{DF}}_{it}+\lambda_{3}{\text{Policy}}_{i}\times {\text{Time}}_{t}+\lambda_{4}{\text{CV}}_{it}+\mu_{i}+\gamma_{t}+\varepsilon_{it}$$

In equation (5), variable *DF* refers to the level of digital finance in the region where the enterprise is located. This study can test the moderating effect of DF through the significance of λ_1_.

The coefficients of *Policy × Time × DF* Table 9 are significant, indicating that DF is the moderating variable for ECRT to affect LCTI. The higher the level of DF in the pilot area, the more effectively ECRT promotes the enhancement of enterprise LCTI. DF, which represents the deep integration of finance and digital technology (Ding et al., 2023), plays a pivotal moderating role in the connection between ECRT and enterprise LCTI.

**Table 9:** Moderating effect of DF.

	(1)	(2)
	LCTI	LCTI
Policy × Time × DF	0.044***	0.039***
	(0.009)	(0.0101)
CV	NO	YES
ID effect	YES	YES
Year effect	YES	YES
Observations	21046	21046
R-squared	0.702	0.789

First, DF diminishes financing barriers and offers financial support for enterprise LCTI. In the context of ECRT policy constraints, the inclusive nature of DF opens doors for numerous green enterprises facing financing constraints, enabling them to access financial markets. This is pivotal in China, where economic disparities can affect access to funds for environmental projects and LCTI. DF democratizes access to finance, mitigates the uneven distribution of capital for environmental initiatives, and combats rent-seeking behaviors, ensuring that more enterprises can secure the necessary funding to accelerate their low-carbon innovations [50].

Second, in the Chinese market, DF lessens the innovation risks associated with LCTI and streamlines the green credit process. Traditional financial channels in China often involve lengthy and complex approval procedures. In contrast, DF leverages advanced digital technologies to expedite credit approvals, enhancing the efficiency of green credit allocations. By optimizing regional credit resource distribution, DF reduces the administrative burden and financial cost for enterprises seeking funding for LCTI projects. DF's risk assessment capabilities can also accurately evaluate the risk levels associated with different low-carbon innovation projects conducted under the constraints of ECRT policy. Consequently, DF assists enterprises in identifying high-risk innovation projects, aligning with the most suitable low-carbon innovation direction, and reducing the risk of LCTI project failure. This, in turn, amplifies their motivation to innovate actively.

### 4.5 Heterogeneity analysis

#### 4.5.1 Heterogeneity in energy-consumption level

We divide the sample into high-energy-consuming enterprises and low-energy-consuming enterprises based on the criteria set by the government, specifically outlined in the *Letter on Clarifying the Implementation of Periodic Policies for Reducing Electricity Costs*. Table 10 shows the regression results of different energy-consuming enterprises. The outcomes indicate that the interaction coefficient for low-energy-consuming enterprises is significantly positive and higher than that of high-energy-consuming enterprises. This discovery underscores the substantial impact of ECRT on enhancing LCTI among low-energy-consuming enterprises.

**Table 10:** Heterogeneity results.

	(1)	(2)	(3)	(4)
	Low energy-consuming	High energy-consuming	Non-SOEs	SOEs
	LCTI	LCTI	LCTI	LCTI
Policy × Time	0.166***	0.074*	0.049	0.183***
	(0.023)	(0.039)	(0.056)	(0.026)
CV	YES	YES	YES	YES
ID effect	YES	YES	YES	YES
Year effect	YES	YES	YES	YES
Observations	15784	5262	14100	6946
R-squared	0.738	0.764	0.712	0.744

In the energy-consuming rights trading market, enterprises operate under bounded rationality and decide to buy or sell rights based on their energy needs and market prices. High energy-consuming enterprises often buy these rights due to their greater energy needs, as the immediate costs of Low-Carbon Technological Innovation (LCTI) can exceed the benefits. Conversely, low energy-consuming enterprises, facing lower costs for these rights and having available funds, are more inclined to invest in LCTI. This reflects a stronger impact of ECRT policy on promoting LCTI among less energy-intensive businesses.

#### 4.5.2 Heterogeneity in enterprise property rights

To test the heterogeneity impact of the implementation of ECRT on LCTI, we divide the sample into state-owned enterprises (SOEs) and non-state-owned enterprises (non-SOEs) based on the nature of enterprise ownership. The regression coefficient of the interaction term for SOEs in Table 10 is higher than that for non-SOEs. This outcome suggests that the positive effect of ECRT on LCTI is more pronounced among SOEs than among non-SOEs. This finding is somewhat counterintuitive given the conventional perception that SOEs may be less responsive to market-based incentives due to their bureaucratic nature. However, in the Chinese context, this can be attributed to the strong policy directives and governmental support that SOEs receive, driving them to comply with national priorities like environmental sustainability and low-carbon development. Therefore, ECRT's impact appears to be more substantial on SOEs, possibly because these entities are more integrated into the national strategy for enhancing LCTI, reflecting a distinctive characteristic of China's mixed economy where state ownership can sometimes accelerate policy implementation. Consequently, compared with non-SOEs, ECRT has a more substantial inducement effect on promoting LCTI among SOEs.

## 5 Conclusions and discussion

### 5.1 Conclusion

Technological innovation serves as the cornerstone for optimizing and upgrading industrial structures. Effectively promoting LCTI among enterprises is a central concern for both academia and governments. Leveraging the ECRT policy implemented in 2017 as a quasi-natural experiment, our study employs the DID method to analyze the policy's impact and influencing mechanisms on enterprise LCTI. We arrive at the following conclusions:

(1) ECRT contributes to enhancing and elevating enterprise LCTI levels by improving their RDC, RDP, and reducing GAC.
(2) ECRT is particularly beneficial for enhancing LCTI among low energy-consuming enterprises and non-SOEs.
(3) DF plays a significant moderating role, meaning that higher DF levels in pilot areas amplify the positive influence of ECRT on enterprise LCTI improvement.

### 5.2 Discussion

The ECRT policy's effectiveness in promoting LCTI supports the Porter Hypothesis. This finding echoes with those of Qi et al. [3], Liu and Sun [31], and Qu et al. [7], who also find that market-driven environmental policies can spur low-carbon innovation. But unlike previous studies focusing on end-of-life regulation tools (e.g., carbon market and low-carbon cities policy), our research sheds light on the impact of upstream control through ECRT, revealing its significant role in enhancing LCTI among enterprises. Our findings also align with Huang et al. [51] and Zhang et al. [37], indicating that increased investment in LCTI fosters technological advancement. This paper goes further by exploring investment as a mechanism for low-carbon innovation under environmental regulation, specifically examining the roles of R&D capital and personnel investments. The existing literature, including studies by Belloc et al. [42] and Tang [43], posits that lowering agency costs can bolster innovation. Our research broadens this concept within the context of environmental economics, enriching the discourse by delving into the less examined mediating influence of decreased green agency costs on innovation. We also contribute to the literature examining the role of digital finance. Literature finds that the development of financial technology can lead to negative outcomes, but corporate social responsibility disclosure this adverse effects [52,53]. Nguyen and Dang [54] reveal that both renewable energy consumption and carbon dioxide emissions negatively affect financial stability, with institutional quality significantly mitigating these effects in more financially stable countries. We shift focus to explore the role of finance in green governance. This change of direction aims to delve deeper into how financial mechanisms can support environmental objectives.

Our study has significant implications for policy-making.

Firstly, it underlines the importance of establishing a unified national market for ECRT within China's environmental policy framework. Our research validates that ECRT, a market-driven approach, effectively stimulates enterprise LCTI. These findings provide empirical support for the induction mechanisms of energy license trading policies on enterprise LCTI within the energy economics domain. Thus, leveraging insights from pilot ECRT projects is essential for developing a robust national system, facilitating a wider adoption of LCTI.

Secondly, there is an urgent need for the government to enhance market-oriented frameworks, including transaction management protocols, technical standards, and operational procedures, to support the ECRT policy fully. As the ECRT transitions from pilot to national scale, efficient trading of energy consumption indicators will benefit enterprises, driving outmoded technologies’ phasing out, improving energy efficiency, and fostering the low-carbon and sustainable transformation of industries. Ensuring the success of the ECRT mechanism requires establishing scientific quota methods, fair and transparent trading, consistent energy consumption measurement, and rigorous regulatory standards.

Thirdly, considering the varied impacts of ECRT on different enterprises, policy designs should acknowledge this heterogeneity. Tailored energy rights allocation plans and supportive measures like R&D subsidies can energize the market, especially for financially constrained firms. Simultaneously, regulatory rigor should increase gradually to avoid negatively impacting competitiveness. For State-Owned Enterprises (SOEs), enhancing the role of LCTI in performance evaluations, possibly through a veto system for environmental compliance, can align their operational goals with broader energy and environmental objectives, fostering sustainable industrial and economic growth.

Our research, while comprehensive, is not without limitations. The lack of comprehensive data constrains our analysis of the ECRT policy's economic and environmental impacts. Specifically, expenditure on low-carbon technology R&D consumes a portion of working capital. But on the plus side, LCTI may help firms reduce compliance costs for policy regulation; and firms with higher LCTI may receive financial transfers from firms that need to decarbonize their energy use. However, such data is unavailable. Additionally, the lack of energy consumption data restricts our ability to evaluate the direct effects of LCTI-driven ECRT on energy savings. This gap in data impedes a nuanced analysis of the policy's efficiency in fostering sustainable energy use. Future research should aim to integrate a broader set of financial indicators to thoroughly assess the cost-effectiveness of LCTI investments under the ECRT framework. Investigating the impact on energy savings, once more detailed data becomes available, will be crucial for understanding the full scope of ECRT's benefits. This approach will help to elucidate the nuanced interplay between market-driven environmental policies and sustainable economic development.

## Ethical approval

Not applicable.

## Data availability

Data are available on request.

## Declarations

### Consent to participate

All authors participated in the process of draft completion. All authors have read and agreed to the published version of the manuscript.

### Consent to publish

All authors agree to publish.

## CRediT authorship contribution statement

**Jingchan Wang:** Investigation, Funding acquisition, Formal analysis. **Wei Chen:** Writing – review & editing, Writing – original draft, Methodology, Conceptualization.

## Declaration of competing interest

The authors declare that they have no known competing financial interests or personal relationships that could have appeared to influence the work reported in this paper.

## Footnotes

1 Xia, Z. (2023, April). Restarting Coal Power to Cope with the Energy Crisis? Differences and Similarities Between China and Europe. China Dialogue. Retrieved from https://chinadialogue.net/zh/4/100998/.

2 Data is obtained from Zhang and Zhou (2021).
